# Supplementary material for: The causal relationship between the human gut microbiota and pyogenic arthritis: a Mendelian randomization study
Source: Front Cell Infect Microbiol. 2024 Nov 26;14:1452480. doi: 10.3389/fcimb.2024.1452480 (PMC11629706; doi:10.3389/fcimb.2024.1452480)
Supplement: Supplementary file 1 [file DataSheet1.zip › mendelian test/ebi-a-GCST90027678.csv_forest.pdf]

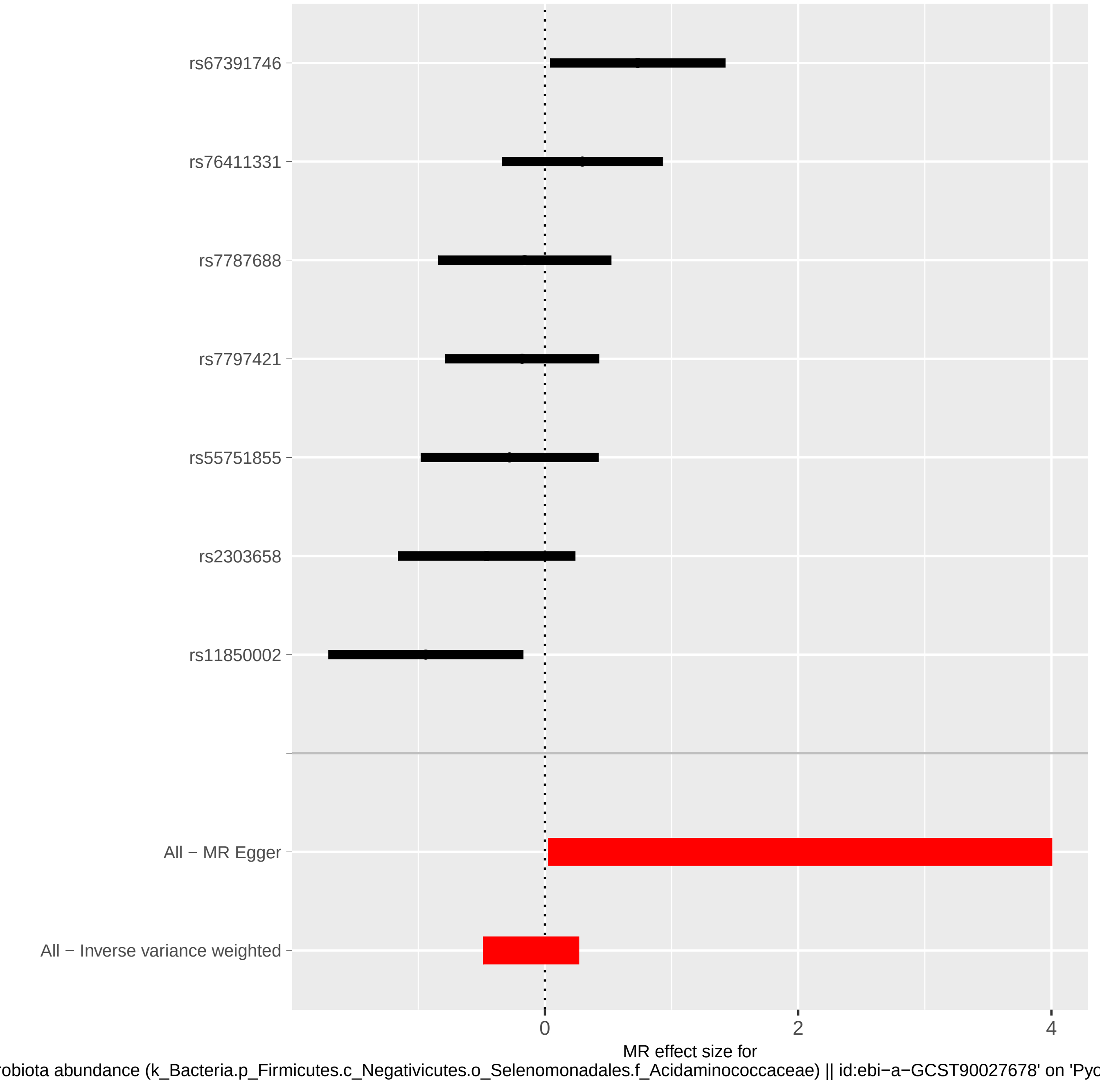
rs67391746
rs76411331
rs7787688
rs7797421
rs55751855
rs2303658
rs11850002
All − MR Egger
All − Inverse variance weighted
0
2
4
MR effect size for
robiota abundance (k_Bacteria.p_Firmicutes.c_Negativicutes.o_Selenomonadales.f_Acidaminococcaceae) || id:ebi−a−GCST90027678' on 'Pyc
